# Germline variants of uncertain significance, their frequency, and clinico-pathological features in a cohort of Sri Lankan patients with hereditary breast cancer

**DOI:** 10.1186/s13104-023-06365-4

**Published:** 2023-06-05

**Authors:** Kawmadi Gunawardena, Nirmala D. Sirisena, Gayani Anandagoda, Nilaksha Neththikumara, Vajira H.W. Dissanayake

**Affiliations:** Department of Anatomy, Genetics and Biomedical Informatics, Faculty of Medicine, University of Colombo, Colombo 8, Sri Lanka

**Keywords:** Breast cancer, cancer phenotype, Germline variants, Hereditary cancer, VUS

## Abstract

**Background:**

Next-Generation Sequencing (NGS)-based testing in cancer patients has led to increased detection of variants of uncertain significance (VUS). VUS are genetic variants whose impact on protein function is unknown. VUS pose a challenge to clinicians and patients due to uncertainty regarding their cancer predisposition risk. Paucity of data exists on the pattern of VUS in under-represented populations. This study describes the frequency of germline VUS and clinico-pathological features in Sri Lankan hereditary breast cancer patients.

**Methods:**

Data of 72 hereditary breast cancer patients who underwent NGS-based testing between January 2015 and December 2021 were maintained prospectively in a database and analyzed retrospectively. Data were subjected to bioinformatics analysis and variants were classified according to international guidelines.

**Results:**

Germline variants were detected in 33/72(45.8%) patients, comprising 16(48.5%) pathogenic/likely pathogenic variants and 17(51.5%) VUS. Distribution of VUS in breast cancer predisposing genes were :*APC*:1(5.8%), *ATM*:2(11.7%), *BRCA1*:1(5.8%), *BRCA2*:5(29.4%), *BRIP1*:1(5.8%), *CDKN2A*:1(5.8%), *CHEK2*:2(11.7%), *FANC1*:1(5.8%), *MET*:1(5.8%), *STK11*:1(5.8%), *NF2*:1(5.8%). Mean age at cancer diagnosis in patients with VUS was 51.2 years. Most common tumour histopathology was ductal carcinoma 11(78.6%). 50% of tumours in patients having VUS in *BRCA1/2* genes were hormone receptor negative. 73.3% patients had family history of breast cancer.

**Conclusions:**

A significant portion of patients had a germline VUS. Highest frequency was in *BRCA2* gene. Majority had family history of breast cancer. This highlights the need to undertake functional genomic studies to determine the biological effects of VUS and identify potentially clinically actionable variants that would be useful for decision-making and patient management.

## Introduction

Worldwide, the most common cancer in women is breast cancer [1]. The incidence of breast cancer in women in Sri Lanka is gradually rising at a rate of 4% per year [2]. Next-generation sequencing (NGS) is increasingly being used to detect germline variants in cancer predisposing genes in hereditary breast cancer. NGS-based multigene panels and clinical exome sequencing are cost- effective methods of testing for variations in many cancer predisposing genes simultaneously. The likelihood of detection of variants increases with the use of NGS techniques [3].

More than ten genes with breast cancer predisposition have been identified over the past 25 years including the high- penetrant tumor-suppressor genes *BRCA1*, *BRCA2*, *PTEN*, *TP53*, *CDH1*, *STK11*, *PALB2* and numerous moderate-penetrant genes like *CHEK2, BRIP1* and *ATM* [4–6].

Variants of uncertain significance (VUS) are DNA variations which have an unknown effect on protein function hence, their association with cancer predisposition risk is uncertain [7]. Frequent usage and rapid expansion of NGS-based testing has led to an increment in detection of VUS. Frequency of VUS among breast cancer patients undergoing NGS-based testing is reported to be around 33–54% [8–10]. However, there is paucity of data on the pattern of VUS in breast cancer predisposing genes in under-represented populations.

When dealing with VUS in the clinical setting, understanding their actionability and providing appropriate genetic counselling poses a challenge to most clinicians [11–13]. In our experience, despite their uncertain significance, such variants create psychological burden to the patients and financial repercussions not only to the patient but to the healthcare system as well due to the sparse and conflicting data on their actionability.

This study aims to describe the frequency and clinico-pathological features of germline VUS identified in a Sri Lankan cohort with hereditary breast cancer and the associated cancer phenotypes in their family members, with a view to building up a genotype-phenotype correlation based on prevailing evidence. We hope this would benefit clinicians in view of deciding management options, arranging family screening and overcoming discrepancies encountered in providing counselling in the context of germline VUS in breast cancer patients.

## Methods

We included 72 consecutive breast cancer affected patients from families with hereditary cancer who underwent germline genetic testing through NGS analysis between January 2015 and December 2021.Their data were maintained prospectively in an anonymized database and analyzed retrospectively. NGS data were subjected to bioinformatics analysis and variants were classified according to American College of Medical Genetics and Genomics and Association for Molecular Pathology standards and guidelines. Clinicopathological data of patients harboring VUS including demographic data, tumour histopathology and receptor status as well as the cancer phenotypes in their first-, second- and third-degree relatives were also analyzed using standard statistical methods. Ethical approval for the study was obtained from the Ethics Review Committee, Faculty of Medicine, University of Colombo [EC-13-182]. Informed written consent was obtained from all patients who underwent germline genetic testing for the participation in the study and for publication of data.

## Results

Germline genetic variants were identified in 33/72 (45.8%) patients. Pathogenic/likely pathogenic variants were detected in 16/33 (48.5%) patients. VUS were identified in 17/33 (51.5%). All the patients harboring VUS were females.

One (5.8%) VUS was novel which was detected in the *CDKN2A* gene and the remaining 16 (94.2%) were reported variants, all were missense variants. The distribution of gene-specific VUS detected in the breast cancer cohort is shown in Table 1. The highest frequency was noted in the high-penetrant *BRCA2* gene 5 (29.4%), followed by moderate-penetrant *ATM* 2 (11.7%) and *CHEK2* 2 (11.7%) genes. Other high-penetrant cancer genes *BRCA1*, *APC*, *STK11* and moderate-penetrant genes *BRIP1*, *CDKN2A*, *FANCI* and *MET* had a similar frequency 1 (5.8%). A VUS was detected in the *NF2* gene, in a young breast cancer patient. *NF2* gene is not reported to be a well-established breast cancer predisposing gene.

**Table 1:** Frequency of gene-specific variants of uncertain significance identified in hereditary breast cancer patients

Gene	Variant ID	Protein change	Frequency	Percentage frequency
*APC*	c.1564 A > G (rs587781692)	p.Met522Val	1	5.8%
*ATM*	c.7502 A > G (rs531617441)	p.Asn2501Ser	2	11.7%
*BRCA1*	c.3392 A > G (rs1555587813)	p.Asp1131Gly	1	5.8%
*BRCA2*	c.784G > A (rs397507393) c.2488 A > G (rs574039421) c.6231G > C (rs541826447) c.521G > A (rs80358747) c.8417 C > T (rs587782785)	p.Ala262Thr p.Asn830Asp p.Lys2077Asn p.Arg174His p.Ser2806Leu	5	29.4%
*BRIP1*	c.3103 C > T (rs45437094)	p.Arg1035Cys	1	5.8%
*CDKN2A*	c.377 A > G	p.Gln126Pro	1	5.8%
*CHEK2*	c.60G > T (rs375507194) c.1501G > A (rs17883172)	p.Gln20His p.Glu544Lys	2	11.7%
*FANCI*	c.3179T > C (rs201376236)	p.Ile1060Thr	1	5.8%
*MET*	c.840G > T (rs1207381066)	p.Arg280Ser	1	5.8%
*STK11*	c.355 A > G (rs545015076)	p.Asn119Asp	1	5.8%
*NF2*	c.1522G > A (rs749326764)	p.Asp508Asn	1	5.8%

The age at cancer diagnosis, tumour histopathological types and receptor status in the breast cancer patients in relation to the gene-specific VUS are shown in Table 2. The highest frequency of patients was observed in the 40–59 years age group (47.1%), followed by the above 60 years age group (29.4%). Youngest patient was aged 28 years and the oldest was 82 years old. Mean age of the cohort was 51.2 years. The most common histopathological type detected was ductal carcinoma. Out of the 14 available histopathology reports, 11 (78.6%) showed ductal carcinoma type. Two (18.2%) among them were detected at the carcinoma in-situ stage while the remaining (81.8%) were invasive type at diagnosis. In the 6 patients with a VUS in the *BRCA* genes whose tumour receptor status reports were available, 50% (3/6) were estrogen (ER) and progesterone (PR) receptor negative. Triple negative tumour was observed only in one patient harboring a *BRCA2* VUS. ER positivity was noted in all other patients with a VUS in the *ATM*, *CDKN2A*, *CHEK2*, *MET*, *STK11* and *NF2* genes.

**Table 2:** Age at cancer diagnosis, tumour histopathology and receptor status in hereditary breast cancer patients in relation to the gene-specific VUS

Gene	Variant ID	Age at diagnosis (years)	Tumour Histopathology	Receptor status				
				ER	PR		HER2	
*APC*	*c.1564 A > G*	63	Invasive ductal	N/A		N/A		N/A
*ATM*	*c.7502 A > G* *c.7502 A > G*	42 39	Invasive ductal Invasive osteoclast like giant cells	+ +		+ +		- -
*BRCA1*	*c.3392 A > G*	51	Invasive ductal	-		-		+
*BRCA2*	*c.784G > A* *c.2488 A > G* *c.6231G > C* *c.521G > A* *c.8417 C > T*	70 28 36 69 70	Invasive ductal Invasive ductal Invasive ductal N/A N/A	N/A - - N/A N/A		N/A - - N/A N/A		N/A - + N/A N/A
*BRIP1*	*c.3103 C > T*	51	Low grade ductal CAIS	N/A		N/A		N/A
*CDKN2A*	*c.377 A > G*	56	Adenocarcinoma	+		-		+
*CHEK2*	*c.60G > T* *c.1501G > A*	51 44	Invasive ductal Papillary	N/A +		N/A -		N/A -
*FANCI*	*c.3179T > C*	46	N/A	N/A		N/A		N/A
*MET*	*c.840G > T*	42	High grade ductal CAIS	+		+		+
*STK11*	*c.355 A > G*	82	Invasive ductal	+		-		-
*NF2*	*c.1522G > A*	31	Invasive ductal	+		+		-

N/A - Data not available; CAIS - Carcinoma-in-situ

The distribution of cancers in close relatives (up to third-degree) of patients with VUS is shown in Table 3. Family history of breast cancer was observed either in first-, second- or third-degree relatives in all patients except in the patients carrying a VUS in the *APC, BRIP1* and *NF2* genes. Family history of gastrointestinal malignancies was observed in patients in whom a VUS was detected in the *APC*, *ATM* and *BRIP1* genes. Family history of leukemia was observed in patients carrying a VUS in the *ATM*, *BRCA1* and *BRCA2* genes.

**Table 3:** Types of cancers in close relatives of patients with hereditary breast cancer in relation to the gene-specific VUS

Gene	Variant ID	Cancers in close relatives		
		First-degree	Second-degree	Third-degree
*APC*	*c.1564 A > G*	Esophageal Melanoma Thyroid (2)		
*ATM*	*c.7502 A > G* *c.7502 A > G*		Colorectal Oral Breast	Breast Colorectal Leukemia
*BRCA1*	*c.3392 A > G*	Breast Leukemia	Oral Cervical	
*BRCA2*	*c.784G > A* *c.2488 A > G* *c.6231G > C* *c.521G > A* *c.8417 C > T*	Breast Ovarian N/A N/A Breast (3) Leukemia Endometrial		
*BRIP1*	*c.3103 C > T*		Colorectal	
*CDKN2A*	*c.377 A > G*	Breast	Breast	
*CHEK2*	*c.60G > T* *c.1501G > A*	Breast (3) Breast		
*FANCI*	*c.3179T > C*	Breast		
*MET*	*c.840G > T*	Breast (2)	Breast	Breast
*STK11*	*c.355 A > G*	Breast (2) colon		
*NF2*	*c.1522G > A*		Testicular	

The number of individuals is stated within brackets if more than one family member is affected

N/A- Data not available

On follow up, the first patient with *ATM* variant (rs531617441) developed stage 1 endometrial cancer after four years. Her mother also developed breast cancer at the age of 80 years. The patient with *NF2* variant (rs749326764) developed liver and vertebral metastasis after one year despite surgical and hormonal therapy and is currently receiving chemotherapy with poor response. The patient with *CHEK2* variant (rs375507194) passed away due to progression of her breast cancer after 2 years. All the other patients are on regular follow up and doing well.

## Discussion

In this study, the frequency of germline VUS was 51.5%, which is in keeping with data from previous studies [8–10]. The highest number of VUS were detected in the *BRCA2* gene. Similar findings were observed in published studies [14–16]. Similar to the findings in this cohort, a relatively high frequency of VUS in the *ATM* gene was reported in several previous studies [14–16]. In contrast to our findings, other studies report a lower frequency of VUS in the *CHEK2* gene [14–16]. This may be attributed to the small sample size in this study. An interesting finding is that though we detected a VUS in the *NF2* gene, no previous studies had reported any VUS in this gene in association with breast cancer [14–16].

The observation of the highest frequency of patients with the age of cancer diagnosis in the 40–59-year age group rather than in the age group over 60 years, contrasts with the observation in a recent Sri Lankan study done based on the national breast cancer database. This observation points towards the hereditary cancer predisposition of individuals in this cohort [2]. Ductal carcinoma was the commonest histopathological type observed and is compatible with the findings from the previous Sri Lankan study [2].

It is well established that variants in the *APC* gene are implicated in the regulation of the intracellular level of beta-catenin through the Wingless/Wnt signal transduction pathway and have been implicated in carcinogenesis through loss of tumour suppressor activity [17]. *APC* gene variants have therapeutic implications as well by imparting chemotherapy resistance highlighting the importance of detecting *APC* variants in cancer patients [18, 19]. The contribution of *APC* variants in colorectal cancer is also well established. The overall *APC* gene variation rate in breast cancer patients ranges between 0.4 and 18% [20, 21]. In this cohort, the patient harboring the *APC* gene VUS at *c.1564 A > G* had a significant family history of esophageal cancer, melanoma and thyroid cancer in first degree relatives. *APC* variants are considered to contribute to esophageal cancer [22–26] and melanoma [27, 28] and are known to have prognostic and therapeutic implications as well. Previous studies have shown thyroid cancer incidence to be increased in patients with *APC* variants [29, 30].

Heterozygosity for *ATM* gene variants increases the risk of development of breast cancer by 2-3-fold compared to the general population [31, 32]. The other cancers seen in the pedigrees of the 2 patients with an *ATM* gene VUS at *c.7502 A > G* were leukemia [33–36] in a third-degree relative and colorectal cancer in second- and third-degree relatives [37, 38]. These cancer types have previously been reported in association with *ATM* gene variants and are considered to have therapeutic implications as well.

*BRCA* gene variants are well recognized in association with breast and ovarian cancers. However, one of the patients with a *BRCA2* gene VUS at *c.8417 C > T* in our cohort had a family history of leukemia and endometrial carcinoma. Several studies have reported leukemia developing after chemotherapy in patients with *BRCA* pathogenic variants but evidence on direct correlation of *BRCA* gene variations with hematological malignancy is not well established [39–42]. Several studies have reported the development of endometrial carcinoma in carriers of *BRCA* gene variants; however, this is largely confounded by Tamoxifen usage in hormone receptor positive breast cancer patients [43–45].

Association of *BRIP1* gene variants with breast cancer is reported [6], however some upcoming controversial data is questioning its significance in the context of breast cancer development [46]. Its predisposition to ovarian cancer is well established in several studies [46, 47]. However, our patient with a VUS in *BRIP1* gene at *c.3103 C > T* did not have a personal or family history of ovarian cancer. Colorectal cancer development in carriers of *BRIP1* gene variants has been reported and a similar occurrence was observed in our patient’s pedigree [48, 49].

The pedigree of the patient with the novel VUS in the *CDKN2A* gene at c.377 A > G clearly depicts a hereditary breast cancer syndrome pattern. Variants in this tumour suppressor gene have been reported previously to be associated with breast cancer development [50, 51].

Germline variations in the *CHEK2* tumour suppressor gene are known to be associated with carcinogenesis [52]. High risk of breast cancer development is observed in individuals carrying *CHEK2* gene variants especially in the context of family history of breast cancer, which is clearly demonstrated in the pedigree of our patient with a VUS in the *CHEK2* gene [53].

Pathogenic variants in the *FANCI*, *MET* and *STK11* have been reported in association with breast cancer [54–56]. Though the evidence for tumour development with *NF2* gene variants is sparse except for the nerve sheath tumours, there is now growing evidence that it may be implicated in the development of several other cancers including breast cancer [57, 58].

This study does not provide data on segregation analysis or functional studies on the biological effects of the VUS. Availability of such data would provide some additional evidence regarding the potential actionability of the variants. Hence, the findings of this study may provide an intriguing line of future research to determine the clinical actionability of the VUS reported herein.

## Limitations

- Small sample size and unavailability of functional data and segregation analysis to assess the biological effects of the VUS.
- Other modifiable risk factors for cancer development were not considered when analyzing the data pertaining to each individual.
- Unavailability of data in few patients pertaining to tumour histopathology, hormone receptor status and family history of cancer.

## Conclusions

The analysis of the cancer phenotypes associated with each VUS including the phenotypic expression in close relatives and comparison with pre-existing data reported before suggests that some of the germline VUS detected in this cohort might be contributing to the cancer development, though currently existing standard classification criteria categorize them as VUS. In the context of VUS, these findings highlight the importance of considering the cancer phenotype of each patient in an individualized manner and incorporating data on cancer expression in other family members in analyzing and interpreting the potential actionability of germline variants in cancer patients which would aid in overcoming to some extent, the discrepancies and conflicts encountered in the process of clinical decision-making.

## Data Availability

The datasets generated and analyzed during the current study are not publicly available but are available from the corresponding author on reasonable request.
